# Association between coffee consumption and high C-reactive protein levels in Korean adults

**DOI:** 10.1017/S0007114523001241

**Published:** 2023-12-28

**Authors:** Sooyeun Choi, Youjin Je

**Affiliations:** Department of Food and Nutrition, Kyung Hee University, Seoul, South Korea

**Keywords:** Coffee, C-reactive protein, Black coffee, Coffee with sugar/cream, Inflammation

## Abstract

The findings of studies investigating the relationship between coffee consumption and C-reactive protein (CRP) levels have been inconsistent, and few researchers considered the type of coffee. We examined the association between coffee consumption and high CRP levels, using data from the Korea National Health and Nutrition Examination Survey, 2016–2018, with 9337 adults aged 19–64 years. A 24-h diet recall was used to assess diet, including the amount and type of coffee consumed. We classified coffee into black coffee and coffee with sugar and/or cream (non-drinkers, or ≤ 1, 2–3, > 3 cups/d) and used multivariable logistic regression models with high CRP levels (≥ 2·2 mg/l). After the adjustment for potential confounders, 2–3 cups/d of coffee consumption were inversely associated with high CRP levels, compared with no consumption (OR = 0·83, 95 % CI 0·69, 0·99). By type of coffee, the inverse association was stronger in subjects consuming black coffee (OR = 0·61, 95 % CI 0·45, 0·84), while the inverse association was much weaker in those consuming coffee with sugar and/or cream (OR = 0·92, 95 % CI 0·74, 1·14). By sex, the inverse association of 2–3 cups of black coffee was found both in men (OR = 0·65, 95 % CI 0·41, 1·03) and women (OR = 0·55, 95 % CI 0·36, 0·83). More than three cups/d of heavy coffee consumption were not significantly associated with high CRP levels. Our findings indicate that moderate black coffee consumption of 2–3 cups/d is inversely associated with high CRP levels in Korean adults. Further prospective studies are warranted to provide definitive evidence.

With a history from the 14th century^(1)^, coffee is one of the most consumed beverages in the world^(2)^. The daily coffee consumption prevalence of Korean adults increased from 54·6 % in 2001 to 65·7 % in 2011 and from 29·1 % in 2001 to 43·3 % in 2011 for those who drank two or more cups of coffee per d^(3)^. The average daily intake of coffee by Korean adults was 1·78 cups per d in 2017^(4)^. In 2019, the average daily intake of coffee (107·5 g) was the largest among total beverages (314·9 g) in the general Korean population^(5)^. Epidemiological studies have shown that coffee consumption lowers the risk of various chronic diseases including CVD, type 2 diabetes, some cancers and overall mortality^(6–9)^. However, the biological mechanisms that explain the association between coffee consumption and chronic diseases remain unknown.

Chronic inflammation is involved in the pathogenesis of various chronic diseases, including CVD, type 2 diabetes and cancer^(10–12)^. C-reactive protein (CRP) is an acute-phase protein that is produced and secreted by hepatocytes through transcriptional control by pro-inflammatory cytokines^(13)^. High-sensitivity C-reactive protein (hsCRP) is a suitable biomarker for evaluating chronic low-grade inflammation. Dietary factors are some of the determinants of inflammatory biomarkers^(14)^. Coffee compounds, such as chlorogenic acid and caffeic acid, have strong antioxidant capacity both *in vivo* and *in vitro* ^(15,16)^. Coffee extracts, including caffeine and kahweol, may relieve inflammation in animals^(17–20)^. Coffee consumption is inversely associated with risk of CVD^(21)^, certain types of cancer^(22–24)^, Parkinson’s disease^(25)^, depression^(26)^ and mortality^(27)^, which have been also associated with higher CRP levels^(28–35)^. Taken together, coffee consumption and inflammatory markers such as CRP levels appear to be related.

Although an inverse association between coffee consumption and inflammatory diseases has been reported^(36–38)^, results suggesting an association between coffee consumption and CRP levels have been inconsistent^(39–55)^. Classifying coffee types is important for conducting studies based on coffee consumption that considers only coffee constituents. Instant coffee mix, containing sugar and/or non-dairy creamers, accounts for a large portion of the Korean coffee market^(3)^. Non-coffee constituents in the instant coffee mix may offset the potential protective effects of coffee. The consumption of instant coffee mix was positively associated with metabolic syndrome^(56)^, weight gain and insulin resistance^(3)^. However, a few studies classified the coffee variety^(39,43,44,51)^, whose results were conflicting, and Korean studies on this topic did not carry out analyses considering coffee types across coffee consumption categories^(41,42,53)^. In addition, the overall effect of coffee intake on health is still unclear. Heavy coffee consumption may be associated with an increased risk of CVD^(57)^. A significant amount of diterpenes in unfiltered coffee may affect the LDL receptor and lead to extracellular accumulation of cholesterol^(58)^.

Therefore, we aimed to evaluate the association between coffee consumption and high CRP levels in Korean adults by considering coffee type, using the data from the Korea National Health and Nutrition Examination Survey (KNHANES).

## Materials and methods

### Study population

We conducted this study based on data from the seventh period (2016–2018) of KNHANES, which is a cross-sectional survey representing the South Korean population. For the KNHANES, which was performed by the Korea Centers for Disease Control and Prevention (KCDC) under the Korean Ministry of Health and Welfare, a stratified, multistage, clustered and probability sampling design was used to collect data representing non-institutionalised civilians in Korea. The KNHANES includes three parts: a health interview, health examination and nutrition survey. The KNHANES methods were previously described in detail^(59)^.

Data were collected from 24 269 participants in 2016 (*n* 8150), 2017 (*n* 8127) and 2018 (*n* 7992). Among them, we included 20 166 participants who completed all three parts (health interview, health examination and nutrition survey) in this study. Then, we excluded the following participants who were not suitable for this study in turn: 8459 subjects who were < 19 or ≥ 65 years old; 670 subjects who self-reported diagnosis of stroke, myocardial infarction/angina, renal failure or cancer; seventy-six subjects who were pregnant; 219 subjects who had missing information on hsCRP levels; 232 subjects who had no fasting status at blood test (< 8 h); 108 subjects who had extreme total energy consumption per d (≤ 500 or > 6000 kcal/d); one subject who drank extremely large amount of coffee per d (> 100 g/d coffee powder); and 1064 subjects who had missing information on alcohol consumption, smoking status or physical activity. We included 9337 subjects (2994 in 2016, 3084 in 2017 and 3259 in 2018), including 5297 women and 4040 men in this study.

**Fig. 1.**
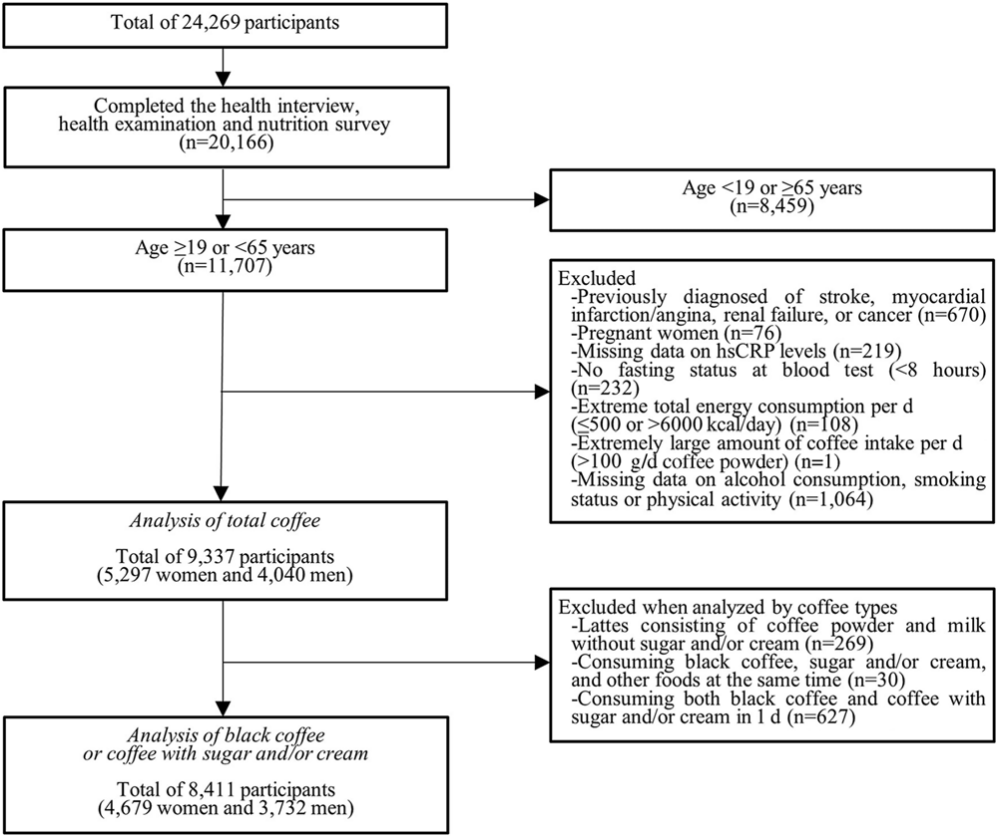
Study participants included in the study after the exclusion criteria. hsCRP, high-sensitivity C-reactive protein.

When analysed by coffee types, the following subjects were additionally excluded from the study population in turn: 269 subjects who drank lattes consisting of coffee powder and milk without sugar or cream because the number of the subjects was too small to be included in the analyses by coffee types; thirty subjects who consumed black coffee, sugar and/or cream, and other foods at the same time because we could not determine whether sugar and/or cream was added to black coffee or other foods; and 627 subjects who consumed both black coffee and coffee with sugar and/or cream in 1 d. We included 8411 subjects (2729 in 2016, 2730 in 2017 and 2952 in 2018), including 4679 women and 3732 men, in the analyses by coffee types. A flow chart of the selection criteria for eligible study population is shown in Fig. 1. Informed consent was obtained from each person in the survey, and the formal ethics approval for the KNHANES dataset was provided by the Centers for Disease Control and Prevention’s Institutional Review Board. (IRB no. 2018–01–03-P-A).

### Assessment of coffee consumption

We used the 24-h recall data from the KNHANES to assess coffee consumption. We used the intake for the tertiary food code. The intake for the tertiary food code is obtained in KNHANES by calculating the conversion coefficient based on the reference solid content because a risk of misinterpretation of intake exists by simply adding the intakes of foods in different states. In the KNHANES, the reference solid food for the intake of the tertiary food code of coffee is in the form of coffee powder. One instant black coffee powder (2·75 g) is defined as the dry weight of one cup of coffee. We converted > 0 to 2·75 g, > 2·75 to 8·25 g and > 8·25 g to ≤ 1 cup, > 1 to 3 cups (2–3 cups) and > 3 cups, respectively.

We classified coffee into black coffee and coffee with sugar and/or cream. The amount of sugar and/or cream is included in the intake for the tertiary food code of coffee with sugar and/or cream. Therefore, we investigated the coffee powder content (%) of each coffee with sugar and/or cream product so that we included only the amount of coffee powder in the coffee intake. If the name of the coffee product was not presented or we could not find the coffee powder content (%) of the coffee product, we used the median value of the coffee powder content (%) of the remaining coffee products.

### Assessment of C-reactive protein levels

The hsCRP level was tested in the KNHANES by Immunoturbidimetry using a Roche Cardiac C-reactive Protein High Sensitive (Roche)^(42)^. In asymptomatic adults, the CRP level of 2·2 mg/l or above was considered elevated^(42,60)^. In the KNHANES 2016, if the hsCRP level was above 20·00 mg/l or less than 0·15 mg/l, then the HE_hsCRP variable, which shows the hsCRP level, was indicated as 20·01 mg/l or 0·149 mg/l, respectively. In the KNHANES 2017–2018, when the hsCRP level was above 20·00 mg/l or below 0·15 mg/l, HE_hsCRP_etc, which was a newly created categorical variable, had a value of > 20·00 or < 0·15, respectively. To ensure data consistency, when the hsCRP level was greater than 20·00 mg/l or less than 0·15 mg/l, we replaced > 20·00 and < 0·15 mg/l of the HE_hsCRP_etc code with 0·149 and 20·01 mg/l of HE_hsCRP code in the data from 2017 and 2018, respectively.

### Confounding variables

Demographic and lifestyle factors such as age, sex, education level, household income, alcohol consumption, smoking status, physical activity, sleep duration and diet quality were obtained using a self-reported questionnaire or by personal interview. Education level was classified into elementary school or lower, middle school or high school, and college or higher. Household income was categorised into quartiles, such as lowest, lower-middle, upper-middle and highest. We multiplied the collected frequency of alcohol consumption by the collected amount of alcohol drank, then calculated the servings of alcohol consumption per d. Then, we categorised alcohol consumption into < 1, 1–< 3 and ≥ 3 servings per d. We grouped smoking status into non-smoker, past smoker and current smoker. We classified physical activity as low and high. We considered high physical activity as at least 75 min of vigorous activity per week, at least 150 min of moderate activity per week or at least 150 min of a combination of vigorous and moderate activity per week (vigorous activity for 1 min was counted as moderate activity for 2 min). We classified sleep duration into < 6, 6 to < 8 and ≥ 8 h per d. We divided diet quality into low and high, which was assessed via a modified diet quality index for Koreans (DQI-K)^(61,62)^. According to cut-point references such as Korean dietary reference intakes (KDRI), the DQI-K points of ‘0 and 1’ or ‘0, 1, and 2’ were assigned to each of the eight factors: daily protein, cholesterol, wholegrain, fruit, vegetable and Na intake, and percent of energy from fat and saturated fat. The sum of the DQI-K point of each factor from each participant ranges from 0 to 9. DQI-K scores of 0–4 and 5–9 reflect high-diet quality and low-diet quality, respectively^(61,62)^.

### Statistical analysis

We used PROC SURVEYFREQ and PROC SURVEYREG (SAS Institute) procedures to estimate the prevalence and mean of demographic and lifestyle factors. We used the PROC SURVEYLOGISTIC procedure to calculate multivariable-adjusted OR and 95 % CI of high CRP levels according to coffee consumption. An OR of less than or greater than 1·0 means that coffee consumption was inversely or positively associated with high CRP levels, respectively. If the 95 % CI includes 1·0, then the OR is not considered statistically significant^(63,64)^. We adjusted model 1 for age (continuous) and sex (women and men). We additionally adjusted model 2 for BMI (kg/m^2^, continuous), daily total energy intake (kcal/d, continuous), education level (elementary school or lower, middle school or high school, and college or higher), household income (lowest, lower-middle, upper-middle and highest), alcohol consumption (> 1, 1–< 3 and ≥ 3 servings/d), smoking status (non-smoker, past smoker and current smoker), physical activity (low and high), sleep duration (< 6, 6 to < 8 and ≥ 8 h/d), DQI-K (low-diet quality (scores of 5–9) and high-diet quality (scores of 0–4)), HDL-cholesterol (mg/dl, continuous), fasting plasma glucose (mg/dl, continuous), TAG (mg/dl, continuous), total cholesterol (mg/dl, continuous), systolic blood pressure (mmHg, continuous), diastolic blood pressure (mmHg, continuous) and leucocyte count (thous/μl). When selecting covariates for a multivariable model, we consulted the previous studies on this topic and the characteristics of the study subjects^(39–44)^. We conducted all statistical analyses with SAS 9.4 software (SAS Institute). We considered a two-tailed *P* value less than 0·05 to indicate statistical significance.

## Results

### General characteristics

Table 1 shows the general characteristics of Korean adults by total coffee consumption. Compared with people in the lowest group (non-drinkers), those in the highest group of total coffee consumption (> 3 cups/d) were more likely to be older and men and to have higher physical activity. Those in the highest category of total coffee consumption also had higher plasma glucose and diastolic blood pressure and lower systolic blood pressure than those in the lowest category. Adults who consumed more total coffee had higher daily energy intake, education level, household income and diet quality, and shorter sleep duration. Frequent total coffee drinkers were likely to be current smokers. Those who drank more total coffee also had higher total cholesterol and leucocyte concentrations.

**Table 1.** Characteristics of study population according to total coffee consumption in Korean adults aged 19–64 years*

	Total coffee consumption								
	Non-drinkers		≤ 1 cup/d		2–3 cups/d		> 3 cups/d		
	Mean or *n*	se or %	Mean or *n*	se or %	Mean or *n*	se or %	Mean or *n*	se or %	*P* §
*n*	2975		2156		3023		1183		
Age (years)	37·10	0·32	45·63	0·30	42·70	0·27	39·12	0·37	< 0·001
Sex (%)									< 0·001
Men	1313	53·74	774	44·98	1423	55·82	530	54·19	
Women	1662	46·26	1382	55·02	1600	44·18	653	45·81	
BMI (kg/m^2^)	23·71	0·09	24·02	0·09	23·89	0·07	24·05	0·14	0·05
Total coffee (cups/d)	0·00	0·00	0·54	0·01	1·81	0·01	4·77	0·06	
Energy intake (kcal/d)	2099·82	21·63	2029·17	22·79	2199·24	20·79	2200·53	32·72	< 0·001
Education level (%)									< 0·001
Elementary school or lower	184	4·08	226	8·21	192	4·80	23	1·31	
Middle school or high school	1459	50·23	1040	49·35	1244	40·34	388	33·76	
College or higher	1331	45·69	888	42·44	1587	54·86	772	64·93	
Household income (%)									< 0·001
Lowest quartile	308	10·31	186	8·23	232	7·33	60	5·47	
Lower-middle quartile	723	23·39	552	24·16	672	21·88	219	17·94	
Upper-middle quartile	908	30·90	707	33·06	998	33·30	357	30·69	
Highest quartile	1032	35·40	710	34·55	1118	37·48	546	45·90	
Alcohol consumption (%)									0·31
< 1 serving/d	2217	72·11	1641	73·16	2214	70·91	866	70·70	
1–< 3 servings/d	462	17·15	330	16·59	536	19·27	209	19·29	
≥ 3 servings/d	296	10·74	185	10·26	273	9·81	108	10·01	
Smoking status (%)									< 0·001
Non-smoker	1905	60·43	1391	58·08	1674	50·02	643	49·58	
Past smoker	592	21·06	384	19·79	619	22·57	226	20·43	
Current smoker	478	18·52	381	22·13	730	27·40	314	29·99	
Physical activity† (%)									< 0·001
Low	1472	45·73	1160	51·06	1656	51·67	574	45·49	
High	1503	54·27	996	48·94	1367	48·33	609	54·51	
Sleep duration (%)									< 0·001
< 6 h/d	434	15·13	279	13·85	362	12·49	180	15·96	
6–< 8 h/d	2132	71·74	1590	73·44	2285	75·85	883	75·65	
≥ 8 h/d	409	13·13	285	12·71	375	11·66	120	8·40	
DQI-K (%)									< 0·001
Low-diet quality	1617	51·08	1278	56·34	1620	50·99	538	43·28	
High-diet quality	1358	48·92	878	43·66	1403	49·01	645	56·72	
HDL-cholesterol (mg/dl)	52·03	0·28	51·97	0·34	51·50	0·27	52·10	0·41	0·42
Plasma glucose (mg/dl)	95·80	0·35	98·97	0·52	98·28	0·45	97·24	0·75	< 0·001
TAG (mg/dl)	133·07	0·35	142·43	3·54	138·99	2·79	135·69	3·64	0·19
Total cholesterol (mg/dl)	188·88	0·77	196·35	0·86	197·07	0·74	197·50	1·11	< 0·001
Systolic blood pressure (mmHg)	114·53	0·30	116·21	0·35	115·41	0·36	113·03	0·45	< 0·001
Diastolic blood pressure (mmHg)	75·93	0·24	77·32	0·26	77·18	0·24	76·33	0·32	< 0·001
Leucocyte (thous/μl)	6·29	0·04	6·20	0·05	6·36	0·04	6·49	0·07	0·004
hsCRP‡ (%)									0·50
Normal	2635	89·30	1937	89·16	2720	90·12	1052	88·39	
Elevated	340	10·70	219	10·84	303	9·88	131	11·61	

DQI-K, a modified diet quality index for Koreans; hsCRP, high-sensitivity C-reactive protein.

* Values are presented as mean and standard errors for continuous variables, and number and weighted % for categorical variables.

† High physical activity was defined as at least 75 min of vigorous activity per week, at least 150 min of moderate activity per week, or at least 150 min of a combination of vigorous and moderate activity per week.

‡ hsCRP was considered elevated if 2·2 mg/l or more.

§ *P*-values obtained from the *χ* ^2^ test for categorical variables and from PROC SURVEYREG procedure for continuous variables.

### Coffee consumption and high C-reactive protein levels

The results of multivariable analyses on the association between coffee consumption and high CRP levels in Korean adults are shown in Table 2. After the multivariable adjustment, we found that adults who consumed 2–3 cups/d of total coffee had 17 % lower odds of high CRP levels than non-drinkers (OR = 0·83, 95 % CI 0·69, 0·99). By type of coffee, the inverse association was stronger in adults consuming black coffee (OR = 0·61, 95 % CI 0·45, 0·84), while the inverse association was much weaker in those consuming coffee with sugar and/or cream (OR = 0·92, 95 % CI 0·74, 1·14). More than three cups/d of heavy coffee consumption was not significantly associated with high CRP levels.

**Table 2.** Multivariable adjusted OR for high CRP levels according to coffee consumption and types in Korean adults aged 19–64 years

	Non-drinkers		≤ 1 cup/d		2–3 cups/d		> 3 cups/d	
Coffee type	OR	95 % CI	OR	95 % CI	OR	95 % CI	OR	95 % CI
Total coffee								
No. of subjects	2975		2156		3023		1183	
No. of cases	340		219		303		131	
Model 1*	1·0	Ref.	0·97	0·78, 1·21	0·87	0·73, 1·04	1·08	0·86, 1·36
Model 2†	1·0	Ref.	0·90	0·72, 1·13	0·83	0·69, 0·99	0·94	0·73, 1·20
Black coffee								
No. of subjects	2975		636		990		603	
No. of cases	340		58		78		58	
Model 1*	1·0	Ref.	0·91	0·66, 1·28	0·66	0·49, 0·88	0·91	0·67, 1·24
Model 2†	1·0	Ref.	0·81	0·56, 1·16	0·61	0·45, 0·84	0·77	0·54, 1·10
Coffee with sugar and/or cream								
No. of subjects	2975		1368		1621		218	
No. of cases	340		144		181		31	
Model 1*	1·0	Ref.	1·02	0·78, 1·32	0·96	0·78, 1·19	1·35	0·86, 2·13
Model 2†	1·0	Ref.	0·97	0·74, 1·26	0·92	0·74, 1·14	1·19	0·74, 1·90

CRP, C-reactive protein; Ref., reference; DQI-K, a modified diet quality index for Koreans.

* Model 1 was adjusted for age and sex.

† Model 2 was adjusted for age, sex, BMI, daily total energy intake, education level, household income, alcohol consumption, smoking status, physical activity, sleep duration, DQI-K, HDL-cholesterol, fasting plasma glucose, TAG, total cholesterol, systolic blood pressure, diastolic blood pressure and leucocyte count.

Table 3 shows the association between coffee consumption and high CRP levels in Korean adults by sex. After the multivariable adjustment, we found that men who consumed 2–3 cups/d of total coffee had 25 % lower odds of high CRP levels than non-drinkers (OR = 0·75, 95 % CI 0·58, 0·98), while the inverse association with 2–3 cups/d of total coffee was weaker in women (OR = 0·91, 95 % CI 0·70, 1·19). By type of coffee, 2–3 cups of black coffee was inversely associated with high CRP levels in men (OR = 0·65, 95 % CI 0·41, 1·03) and women (OR = 0·55, 95 % CI 0·36, 0·83), and the association seems slightly stronger in women. More than three cups/d of heavy coffee or consumption of coffee with sugar and/or cream were not significantly associated with high CRP levels both in men and women.

**Table 3.** Multivariable adjusted OR for high CRP levels according to coffee consumption and types in Korean adults aged 19–64 years by sex

	Non-drinkers		≤ 1 cup/d		2–3 cups/d		> 3 cups/d	
Coffee type	OR	95 % CI	OR	95 % CI	OR	95 % CI	OR	95 % CI
Men								
Total coffee								
No. of subjects	1313		774		1423		530	
No. of cases	172		85		160		73	
Model 1*	1·0	Ref.	0·87	0·63, 1·20	0·83	0·65, 1·05	1·07	0·78, 1·47
Model 2†	1·0	Ref.	0·78	0·55, 1·10	0·75	0·58, 0·98	0·89	0·63, 1·24
* *Black coffee								
No. of subjects	1313		198		354		248	
No. of cases	172		17		34		28	
Model 1*	1·0	Ref.	0·67	0·37, 1·23	0·69	0·45, 1·05	0·81	0·51, 1·29
Model 2†	1·0	Ref.	0·58	0·30, 1·12	0·65	0·41, 1·03	0·73	0·43, 1·23
Coffee with sugar and/or cream								
No. of subjects	1313		548		933		138	
No. of cases	172		61		108		23	
Model 1*	1·0	Ref.	0·90	0·63, 1·31	0·85	0·65, 1·12	1·31	0·78, 2·19
Model 2†	1·0	Ref.	0·82	0·55, 1·22	0·77	0·57, 1·05	1·05	0·62, 1·79
Women								
Total coffee								
No. of subjects	1662		1382		1600		653	
No. of cases	168		134		143		58	
Model 1*	1·0	Ref.	1·11	0·84, 1·45	0·93	0·73, 1·20	1·09	0·76, 1·56
Model 2†	1·0	Ref.	1·07	0·81, 1·43	0·91	0·70, 1·19	1·01	0·69, 1·49
Black coffee								
No. of subjects	1662		438		636		355	
No. of cases	168		41		44		30	
Model 1*	1·0	Ref.	1·18	0·80, 1·75	0·63	0·43, 0·94	1·05	0·67, 1·66
Model 2†	1·0	Ref.	1·06	0·69, 1·64	0·55	0·36, 0·83	0·82	0·51, 1·33
Coffee with sugar and/or cream								
No. of subjects	1662		820		688		80	
No. of cases	168		83		73		8	
Model 1*	1·0	Ref.	1·17	0·85, 1·62	1·17	0·87, 1·58	1·29	0·51, 3·24
Model 2†	1·0	Ref.	1·16	0·83, 1·62	1·13	0·82, 1·55	1·32	0·51, 3·42

CRP, C-reactive protein; Ref., reference; DQI-K, a modified diet quality index for Koreans.

* Model 1 was adjusted for age.

† Model 2 was adjusted for age, BMI, daily total energy intake, education level, household income, alcohol consumption, smoking status, physical activity, sleep duration, DQI-K, HDL-cholesterol, fasting plasma glucose, TAG, total cholesterol, systolic blood pressure, diastolic blood pressure and leucocyte count.

## Discussion

This study examined the association between coffee consumption and high plasma CRP concentrations in a total of 9337 Korean adults, using data from the KNHANES, a large nationally representative study of Korean population. In the current study, we found that 2–3 cups/d of coffee consumption was inversely associated with high CRP levels. By type of coffee, 2–3 cups/d of black coffee was associated with 39 % lower odds of high CRP levels, and the inverse association with moderate black coffee consumption was found both in men and women. However, there was no significant association of coffee with sugar and/or cream and high CRP levels.

Consistent with the results of cross-sectional studies^(39,40,43,46,48,49,51–54)^ and one meta-analysis study^(45)^, we observed inverse associations between coffee consumption and high plasma CRP concentrations. Coffee consumption may be associated with several health conditions in a J-shaped relationship^(58)^. In one meta-analyses, coffee consumption of 2·5 cups per d produced the largest reduction in CVD mortality^(65)^. One cohort study found a non-linear association of coffee consumption with CVD and all-cause mortality, with the largest risk reduction seen at 2–3 cups/d^(66)^. In one meta-analysis, an inverse relationship was identified between coffee consumption and risk of CVD, which was the lowest at 3–5 cups/d^(67)^. In other words, heavy coffee consumption does not further reduce risk of CVD or all-cause mortality compared with moderate coffee consumption. The certain coffee intake level of protective effect varies from study to study, which may be due to the different amounts considered one cup of coffee set in each study. These results of previous studies on the benefits of coffee at the specific level of coffee intake are consistent with our results.

For moderate black coffee consumption of 2–3 cups per d, protective effects may outweigh adverse effects, whereas for heavy black coffee consumption of more than three cups/d, adverse effects may offset protective effects^(67)^. Coffee contains various bioactive compounds, including caffeine, chlorogenic acid, caffeic acid and kahweol, which can exert protective effects on health via anti-inflammatory and antioxidant capacities^(15–20)^. Coffee also contains bioactive components that can adversely affect health. Coffee with a significant amount of diterpenes, with known lipid-raising effects, may be associated with higher LDL-cholesterol levels^(43)^. Diterpenes in unfiltered coffee may affect the LDL receptor and lead to extracellular accumulation of cholesterol. However, there is no evidence that long-term coffee consumption is associated with dyslipidemia^(58)^. In addition, caffeine increases blood pressure acutely by antagonising adenosine A1 and A2A receptors^(67)^. However, tolerance develops rapidly, and the effects of caffeine on long-term blood pressure control are minimal^(66)^. Another reason that the lower risk of elevated CRP levels disappears with heavy coffee consumption may be the residual confounding effects of smoking, which is an important confounder for the association of coffee consumption with CVD and may bias the relative risk upwards^(65,66)^.

The mechanisms supporting the inverse association between coffee intake and CRP levels may be explained as follows: caffeine, a major component of coffee, has relieved inflammation or reduced CRP levels in several animal studies. Caffeine could decrease plasma CRP levels^(68)^ and could mitigate pulmonary inflammation^(17,18,20)^ and retinal inflammation by showing its anti-inflammatory and neuroprotective effects via A2A adenosine receptor signalling^(69)^. Another main ingredient in coffee, kahweol, showed anti-inflammatory ability *in vivo* through the inhibition of COX-2 and iNOS expression at inflammatory sites^(19)^. Additionally, the chlorogenic acid in coffee may decrease the synthesis of inflammatory mediators by lowering the expression of pro-inflammatory cytokine genes, inhibiting protein tyrosine phosphatase 1B and regulating NF-κB activation^(58)^. Plasma chlorogenic acid exhibited an inverse relationship with CRP levels^(70)^. In addition, the melanoidins produced during coffee bean roasting could reduce chronic inflammation by increasing anti-inflammatory mediators^(71)^. However, no experimental studies have determined the association between coffee as a whole and CRP levels directly. Therefore, the underlying mechanisms need to be further examined in future studies.

In this study, the association between coffee with sugar and/or cream and high CRP levels was not significant overall. The inflammatory effect of added sugar may offset the anti-inflammatory effect of coffee. The findings of previous studies on the positive association between sugar intake and high CRP levels support this^(72,73)^. A review study has reported that added sugar may need to be included as a potential confounder in studies on the association between coffee consumption and health by sufficient evidence^(74)^.

Moderate black coffee consumption showed a strong inverse association with high CRP levels, especially in women (OR = 0·55, 95 % CI 0·36, 0·83), which is consistent with the previous studies^(52–54)^. A previous study reported that this association might be caused by female-specific anti-inflammatory effects of coffee^(52)^. However, previous studies with large sample sizes have shown a significant inverse association between coffee consumption and CRP levels in both men and women^(39,43)^. We did not find a significant inverse association between black coffee consumption and high CRP levels in men, but the association between 2–3 cups/d of black coffee consumption and high CRP levels tended to be inverse in men (OR = 0·65, 95 % CI 0·41, 1·03). Additionally, the number of men who drank black coffee was relatively small compared with that of women. Thus, there may not be a sex difference in the association between coffee consumption and high CRP levels.

Our study has some strengths and limitations. To the best of our knowledge, this is the first study using the KNHANES to examine the relationship between coffee consumption and high CRP levels according to the coffee type, separately in men and women. Because this study was based on nationally representative data of the Korean population, our findings can be generalised to Koreans. To reduce the influence of diet and lifestyle factors as potential confounders, we tried to adjust for dietary quality using DQI-K, as well as health behaviours such as alcohol consumption, smoking status, physical activity and sleep duration. Nonetheless, our findings should be interpreted with caution because our study design was cross-sectional, so the sequence of events cannot be inferred, and causal inferences are limited. Those with morbid conditions with highly elevated CRP levels may have reduced coffee consumption^(75)^, so we excluded participants with diseases such as stroke, myocardial infarction/angina, renal failure or cancer from the study population. In addition, we could not consider the information on other types of coffee (e.g. caffeinated coffee, decaffeinated coffee, Dutch coffee, etc.), and the amount of caffeine in coffee or caffeine intake through the consumption of other foods such as green tea, black tea or energy drink, because of the limited data or the insufficient number of subjects to analyse. Decaffeinated coffee may have anti-inflammatory effects similar to those of caffeinated coffee due to the action of bioactive constituents other than caffeine^(39,43,51)^. Additionally, studies have shown that decaffeinated coffee as well as caffeinated coffee are inversely associated with risk of CVD, cardiovascular mortality and all-cause mortality^(66,76,77)^. Another study reported that the inverse association between coffee consumption and cardiometabolic markers or all-cause mortality was not modified by genetic factors affecting caffeine metabolism^(43,66)^. As our study was conducted based on a single measurement of hsCRP levels, random measurement errors would likely attenuate the findings towards the null. Previous studies that measured diet multiple times during study follow-up recorded relatively stable coffee consumption patterns and indicated that a single measurement of coffee intake may reflect medium- to long-term coffee consumption^(52)^.

Our results differ from those of several previous studies^(41,42,44,47,50,55)^ examining the relationship between coffee consumption and CRP levels. We found that moderate black coffee consumption of 2–3 cups per d was inversely associated with high CRP levels in Korean adults. The differences may be due to analysis by coffee type, selection of study subjects according to strict criteria and adjustment for diet quality using DQI-K. High-sensitivity CRP levels were measured in the KNHANES. The findings of one meta-analysis suggested that hsCRP was more sensitive than standard CRP in demonstrating the anti-inflammatory capacity of coffee^(45)^.

In conclusion, moderate black coffee consumption of 2–3 cups per d was inversely associated with high CRP levels in Korean adults. These findings contribute to suggesting the pathobiological mechanisms supporting the inverse association between coffee consumption and the risk of chronic diseases such as CVD. Further prospective studies are necessary in large populations to determine the association between various types of coffee consumption and high CRP levels.
